# Satellite Observation Mission Resource Scheduling Based on Dynamic Coalition Algorithm

**DOI:** 10.3390/s25226817

**Published:** 2025-11-07

**Authors:** Shijie Zhai, Tinghua Zhang, Hao Chen

**Affiliations:** Department of Electronic and Optical Engineering, Space Engineering University, Beijing 101416, China; 15810887677@163.com (S.Z.); 18317921185@163.com (H.C.)

**Keywords:** dynamic coalition algorithm, satellite observation mission, resource scheduling, five-dimensional evaluation, simulated annealing optimization

## Abstract

This study was conducted in response to the challenges posed by the heterogeneity of ground station resources and the dynamic nature of tasks in satellite observation missions. To combat these issues, we propose a resource scheduling method based on a dynamic coalition algorithm. The method involves constructing a five-dimensional evaluation system including spatial proximity, energy sufficiency, equipment integrity, load balancing, and continuous observation capability, which is combined with an improved simulated annealing algorithm to achieve global optimization of the coalition structure. Then, an energy allocation strategy based on demand is designed to enhance system sustainability. An experiment comparing the greedy, particle swarm, genetic, and simulated annealing algorithms was conducted. The results showed that the task completion rate of the dynamic coalition algorithm reached 93.8%; the resource utilization rate was 85.7%; the energy consumption standard deviation was 18.7; and the convergence speed was 45 iterations for the proposed method. These results were significantly better than those of other algorithms used for comparison. The innovative aspects of this study include ① a dynamic energy allocation model based on normalized priority; ② a simulated annealing optimization framework with hybrid neighborhood operations; and ③ the deep integration of multi-dimensional evaluation metrics and dynamic coalition construction mechanisms. This research provides theoretical support and technical solutions for task scheduling in wireless sensor networks under complex dynamic scenarios.

## 1. Introduction

Wireless sensor networks, as key enablers of next-generation intelligent sensing and information exchange technologies, have demonstrated significant advantages in fields such as environmental monitoring, military reconnaissance, and disaster warning. Leveraging the collaborative sensing and resource-sharing capabilities of distributed nodes can achieve efficient observation and decision support for complex dynamic scenarios [1]. However, in three-dimensional space observation tasks, sensor nodes must simultaneously meet the spatiotemporal and resource constraints and quality of service requirements of multiple objectives, posing a significant challenge for the network’s task allocation mechanism [2,3]. Particularly when wireless sensor networks are deployed in space applications (such as satellite observation or drone swarms), communication links between nodes encounter dynamic environments akin to multi-orbit satellite networks. These networks encompass signal attenuation, Doppler effects, and interference issues. In such scenarios, precise calculation of the link budget is crucial to ensure the reliability of communication [4]. This involves a comprehensive analysis of signal gains and losses to determine the link margin—the surplus capacity beyond minimal requirements that compensates for unpredictable channel degradation. The construction of an efficient and stable collaborative observation coalition has become a core issue under the combined constraints of heterogeneous ground station resources, limited energy availability, and dynamic mission requirements to enhance the operational effectiveness of wireless sensor network missions [4].

Traditional task allocation methods are often based on static resource allocation strategies or single optimization objectives (such as minimizing energy consumption or maximizing coverage). This makes these methods difficult to adapt to spatiotemporal correlations and different priorities assigned to tasks in dynamic observation scenarios. In particular, in scenarios such as satellite observation and mobile target tracking, differences in ground station control capabilities, the dynamic characteristics of energy consumption, and the time-sensitive requirements of observation tasks necessitate a dynamic collaborative mechanism that balances resource utilization, task completion rates, and system robustness. However, existing research often overlooks the challenges of integrating multi-layer satellite systems. As Höyhtyä et al. (2024) pointed out, deploying MLSS results in complex issues such as spectrum management, inter-layer coordination, and interference control [5]. Examples include interference avoidance when GEO and NGSO satellites coexist in the same frequency band and dynamic routing optimization to balance latency and jitter. In recent years, dynamic coalition theory has provided novel insights into task coordination in multi-agent systems. By dynamically constructing and optimizing temporary collaborative organizations, it effectively integrates dispersed resources and rapidly responds to mission requirements. However, existing research inadequately evaluates the multi-dimensional attributes of nodes during federation formation and fails to consider dynamic energy consumption allocation mechanisms and long-term load balancing [5]. This results in limited resource allocation efficiency and a constrained network lifecycle. Furthermore, traditional approaches often neglect the sensitivity analysis of communication links. For instance, the selection of digital modulation schemes (e.g., PSK, DPSK, QAM, and OFDM) significantly impacts the link budget and margin, thereby determining system reliability and spectral efficiency in dynamic environments. In multi-orbit systems (e.g., LEO, MEO, GEO), various modulation schemes exhibit vastly different sensitivities to link margins. For instance, OFDM offers high spectral efficiency in dynamic environments but requires higher signal-to-noise ratios, while PSK is more robust at the cost of data rate. Existing research primarily focuses on static link budgets, lacking in-depth exploration of modulation scheme sensitivity in multi-orbit dynamic scenarios [6]. Studies fail to integrate error correction codes (e.g., LDPC and Reed–Solomon codes) to optimize link performance.

In this study, we address the aforementioned issues by proposing a dynamic coalition task allocation model for wireless sensor networks in three-dimensional observation scenarios. By defining multi-dimensional matching rules between ground station resource matrices and task demand vectors and combining spatial constraints, energy consumption models, and task priority mechanisms, we establish a dynamic coalition construction method based on five-dimensional evaluation indicators. Inspired by the MLSS architecture, this model introduces the concept of Inter-Layer Links, enabling the dynamic routing of task data between heterogeneous nodes to simulate collaboration between satellite layers (e.g., LEO-GEO backhaul). Such collaboration enhances coverage and resilience. Based on this method, an improved simulated annealing algorithm was designed to achieve global optimization of the coalition structure. Energy demand-based allocation strategies and load balancing mechanisms were employed to enhance the network’s sustainability. The innovative nature of this study is primarily reflected in the following aspects: ① A task execution model based on normalized priority and dynamic energy allocation is proposed to achieve resource allocation prioritization for high-value tasks, and ② a multi-dimensional evaluation system is established, integrating spatial proximity, energy abundance, equipment integrity, load balancing, and continuous observation capability. Inspired by multi-orbit link budget analysis, this system quantifies the impact of modulation schemes and error correction codes on link margins [5]. ③ A simulated annealing optimization framework with hybrid neighborhood operations is designed to effectively balance local search and global exploration capabilities [7]. The results of simulation experiments demonstrate that the proposed method significantly outperforms traditional algorithms in terms of the task completion rate, resource utilization efficiency, and algorithm convergence [8].

The overall structure of this thesis is as follows: Section 1 serves as the introduction. Section 2 constructs a priority scheduling model for satellite constellation tasks, defining the resource matrix and constraint conditions. Section 3 proposes a dynamic ground station coalition model and elaborates on its lifecycle stages. Section 4 designs a five-dimensional evaluation metric and employs an improved simulated annealing algorithm to optimize the selection of coalition members. Section 5 compares algorithmic performance through simulation experiments, validating metrics such as mission completion rate and resource utilization; Section 6 summarizes innovations and discusses future research directions.

## 2. Establishing a Mission Priority Scheduling Model for Satellite Constellations

Various satellite observation tasks are received and prioritized based on urgency. The ground station energy state formula is used to determine the energy status capability of ground stations to execute corresponding satellite observation tasks [6]. The observation energy of ground stations for satellites is determined using the ground station energy state function, comprehensively considering satellite facilities and ground station communications, which is calculated using the following formula:

(1) $$E_{ij\;}=\beta_{1\;}\cdot f(\theta_{i})\cdot d_{j}\;\;\;+\beta_{2}\;\cdot g(\theta_{i})\cdot V_{j}\;\;\;$$

Here, $E_{ij\;}$ represents the energy consumed by node *i* to execute task *j*. $\beta_{1\;}$ is the energy consumption weighting coefficient for node-type tracking. $f(\theta_{i})$ is the energy consumption coefficient for node-type tracking. $\theta_{i}$ denotes the type of node *i*; $d_{j}$ represents the duration of task *j*; $\beta_{2}$ is the transmission energy consumption weight coefficient for node type; $g(\theta_{i})$ is the transmission energy consumption coefficient for node type; and $V_{j}$ is the total data volume required for transmission by task *j*.

(2) $$f(\theta_{i})=\;\;\;k_{D}\cdot \log(D+1)+k_{f_{c}}\cdot \frac{f_{c}}{f_{c0}}+k_{\sigma }\cdot \frac{\sigma_{0}}{\sigma }\times (1+k_{s}\cdot A_{env})$$

Here, $k_{D}$ denotes the weighting coefficient for the distance term, which is used to adjust the importance or proportion of the distance factor within the total score $f(\theta_{i})$; D represents the node antenna aperture; $k_{f_{c}}$ indicates the weighting coefficient for the frequency term; $f_{c}$ signifies the tracking carrier frequency; and $f_{c0}$ denotes the reference carrier frequency. $k_{\sigma }$ represents the weighting coefficient for the stability factor, adjusting its importance to the overall score; $\sigma_{0}$ represents tracking accuracy; σ represents baseline accuracy; $k_{s}$ represents the deployment stability coefficient; and $A_{env}$ represents the environmental attenuation coefficient.

(3) $$g(\theta_{i})=k_{link}\cdot T(link)+k_{Rb}\cdot \frac{R_{b}}{R_{b0}}+k_{mod}\cdot M(mod)\times (1+k_{int}\cdot I_{env})$$

Here, $k_{link}$ denotes the transmission link type, T(link) represents the link performance metric (indicating performance parameters related to the communication link), link signifies the communication link itself, $k_{Rb}$ indicates the weighting coefficient for the data transmission rate component, $R_{b}$ denotes the data transmission rate, and $R_{b0}$ represents the baseline rate. $k_{mod}$ denotes the weighting coefficient for module items, which used to adjust the importance of module performance to the overall score. M(mod) represents module performance metrics, signifying the performance parameters of specific functional modules within the equipment. mod indicates the modulation scheme. $k_{int}$ denotes the interference sensitivity coefficient, and $I_{env}$ represents the environmental interference intensity.

The energy state function of the node ground station determines the potential leader of the dynamic coalition satellites. Ground stations traverse around this leader to perform coalition member expansion [7]. The total energy of each ground station is represented as coalition members’ energy consumption distributed proportionally to their current energy levels against the total mission demand, following these specific allocation rules:

(4) $$E_{all}=\sum e_{i\;}(t)+\sum E_{ij}$$

In the above equation, $E_{all}$ represents the total energy of each ground station; $e_{i\;}(t)$ denotes the remaining energy of node *i* before executing a task; and *t* is the time variable. $E_{ij}$ indicates the energy consumption required for node *i* to perform task *j*; *i* represents the node number; and *j* denotes the task number.

The satellite observation mission can be executed only when $E_{all}\geq E_{j}$. The energy consumption of member node i for the satellite observation mission j is as follows:

(5) $$\Delta e_{i,j}=(\frac{e_{i}(t)}{E_{all}})E_{j}$$

After completing the task, member i’s energy is updated to the following:

(6) $$e_{i}(t+1)=e_{i\;}(t)-[\gamma \cdot \Delta e_{i,j}+(1-\gamma )\cdot \frac{Con_{i}(\theta_{i})\;}{\sum Con_{i}(\theta_{i})\;}\;\;\;\cdot {E_{j}}^{actual}\;]$$

(7) $$Con_{i}(\theta_{i})=\left\{\begin{array}{c} C_{\mathrm{trk\_stat}}\;\;\;(\theta_{i}=1)\\ \begin{array}{l} C_{\mathrm{data\_stat}}\;\;\;(\theta_{i}=2)\\ C_{\mathrm{combo\_stat}}\;\;\;(\theta_{i}=3) \end{array} \end{array}\right.$$

In the formula, $e_{i}(t+1)$ represents the remaining energy of node *i* after executing a task; γ denotes the tuning parameter; $\Delta e_{i,j}$ indicates the energy consumed by node *i* when executing task *j* under condition $E_{all}\geq E_{j}$; $Con_{i}(\theta_{i})$ signifies the actual contribution of node *i*; and ${E_{j}}^{actual}$ denotes the actual total energy consumption of task *j*. $\theta_{i}$ represents the type of *i*-th node, where 1 denotes a telemetry and control station, 2 denotes a data transmission station, and 3 denotes an integrated station. $C_{\mathrm{trk\_stat}}$ represents the tracking capability score, where a higher score indicates stronger capability; $C_{\mathrm{data\_stat}}$ indicates the data transmission capability score, where a higher score signifies greater data transmission capability; $C_{\mathrm{combo\_stat}}$ reflects the composite capability score, which is used to select the optimal coalition leader or member based on the scores.

The tracking station is evaluated based on tracking duration, using a positive weighting for the compliance rate and a negative penalty for the number of interruptions:

(8) $$C_{\mathrm{trk\_stat}}=\left[k_{T}\cdot T_{trk}\cdot P_{\sigma }-k_{N}\cdot \log(N_{int}+1)\right]\cdot \alpha_{task}$$

In the formula, $k_{T}$ represents the weighting coefficient for tracking performance; $T_{trk}$ denotes the duration of successful tracking; $P_{\sigma }\;$ indicates the tracking accuracy compliance rate; $k_{N}\;$ signifies the weighting coefficient for the interruption penalty term; $N_{int}$ reflects the number of tracking interruptions; and $\alpha_{task}$ represents the mission type coefficient.

The data transmission station is based on error-free data volume, with positive weighting for rate compliance and a negative penalty for bit error rate:

(9) $$C_{\mathrm{data\_stat}}=\left[k_{V}\cdot V_{data}\cdot P_{Rb}-k_{BER}\cdot \log(BER+10^{-8})\right]\cdot \beta_{task}$$

Among these, $k_{V}$ represents the weighting coefficient for the data volume metric; denotes the error-free transmitted data volume; $P_{Rb}$ indicates the transmission rate compliance rate; signifies the weighting coefficient for the error penalty term; reflects the average data error rate; and $\beta_{task}$ denotes the data type coefficient.

The integrated station must combine contributions from both “tracking” and “transmission” dimensions, with weighting allocated based on the proportion of functional input:

(10) $$C_{combo}=\omega_{trk}\cdot C_{\mathrm{trk\_sub}}+\omega_{data}\cdot C_{\mathrm{data\_sub}}$$

Here, $\omega_{trk}$ represents the weight coefficient for the tracking capability sub-item, and $\omega_{data}$ denotes the weight coefficient for the data transmission capability sub-item. There are three categories of ground station types: tracking and control stations, data relay stations, and integrated stations. Unlike the other two types, integrated stations possess the capabilities of both tracking and control stations and data relay stations. The above formula represents the contribution capacity of integrated stations.

If $E_{all}\leq E_{j}$, the task is terminated without consuming energy. In the ground station energy update strategy defined above, the higher the task priority, the greater the energy demand. This reflects the allocation of resources to high-value tasks. Energy is allocated proportionally, with energy consumption being proportional to the current energy of each member, incentivizing the selection of high-energy nodes to join the coalition. The station’s energy is updated after each task, affecting the coalition formation strategy for subsequent tasks.

## 3. Dynamic Coalition Model for Satellite Observation Mission Ground Stations

A dynamic coalition consists of a coalition leader and several coalition members. The Dynamic Coalition Law terminates the system and dissolves the coalition upon determining that an event has concluded or related matters have been resolved. The primary function of this form is to coordinate various intelligent sensor nodes within the network, ensuring their effective communication and detection performance while leveraging the advantages of each node [8]. The process of forming a dynamic coalition can generally be divided into four stages: coalition initialization, coalition establishment, coalition operation, and the dissolution phase [9].

**Figure 1:**
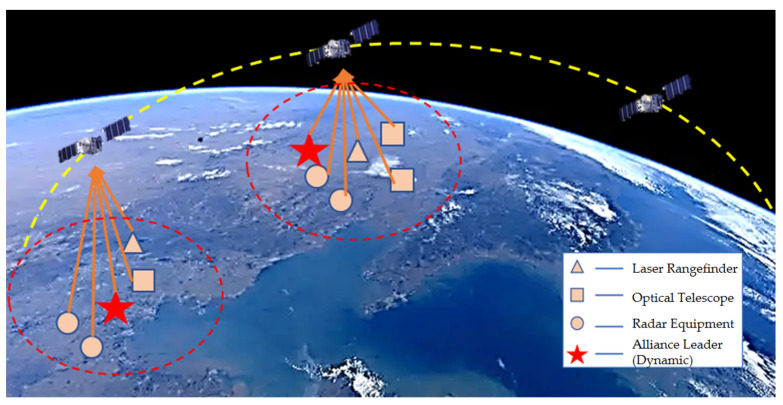
Schematic diagram of dynamic coalition operation.

The lifecycle of a coalition is determined by detection task(s) and specific events. When a detection event occurs and observation zone nodes actively initiate detection tasks, a coalition is established [10]. After the nodes capture the event, they issue a collaborative request to initiate the establishment of a coalition. Through task allocation, dynamic confirmation, and planning, the coalition executes its operational process. Finally, the coalition dissolves upon the conclusion of the detection event. In this process, the most time-consuming and resource-intensive stages are coalition initialization and the operational process [11]. During these phases, the coalition generates a large volume of communication data, including task and feedback confirmations, as well as other types of dynamic authentication information. The specific composition of the dynamic coalition is shown in Figure 1.

Large data volumes necessitate longer transmission times. Furthermore, the waiting time for data in the transmission queue and retransmissions caused by channel instability significantly increases end-to-end transmission latency. This poses significant challenges for observation tasks that require high real-time performance, potentially rendering data “outdated” and losing its value [12]. In wireless communications, data transmission is the primary source of energy consumption. According to classical communication models, node energy expenditure is directly proportional to the volume of transmitted data. Sustained, high-intensity downlink operations rapidly deplete ground station batteries (especially data relay stations), shortening their operational lifespan and compromising the long-term stability of the entire observation network. Rather than passively adapting to these challenges, this paper’s model transforms them into manageable optimization objectives within the system through proactive, forward-looking resource scheduling and coalition-building mechanisms. Through intelligent coalition member selection and load balancing, this model optimizes network throughput and alleviates bottlenecks. Its core principle is collaborative cooperation rather than single-point responsibility. When large-data-volume tasks form a coalition, the coalition leader node (typically a comprehensive station) automatically favors the selection of dedicated data relay stations to join the coalition through its weighted member selection objective function [13]. This creates an efficient “tracking-data transmission separation” load-sharing model: the coalition leader handles satellite tracking, command uplink, and other control tasks, while multiple data relay station members can receive data downlinks in parallel and collaboratively. This approach effectively aggregates the communication bandwidth of multiple stations, decomposing centralized large data streams into parallel sub-streams. This significantly enhances the system’s overall throughput capacity and prevents any single ground station from becoming a performance bottleneck.

The coalition leader node is determined based on its proximity to the transaction processing location. Other coalition member nodes are responsible for observation and analysis within their respective regions and provide descriptions of detailed tasks [14]. The coalition leader node supervises and coordinates the member nodes, integrates and processes the valid information uploaded by all nodes, and transmits it to the aggregation node, thereby concluding the dynamic coalition process within its scope. For mobile target detection processes, dynamic coalition collaborative detection offers higher robustness and better adaptability to complex conditions. It also provides a more comprehensive orientation for acquiring target information. The process is illustrated in the following method:

Step 1: Begin with coalition initialization. The coalition leader that first detects target units within its detection range dynamically assesses and monitors surrounding nodes that are capable of forming a network through methods such as domain-wide broadcasts. The coalition leader broadcasts a coalition formation request to all potential nodes within the detection range and receives real-time status data (including remaining energy, device health, spatial position, and available bandwidth) reported by each node. Based on a five-dimensional evaluation system (spatial proximity, energy sufficiency, device integrity, load balancing, and sustained observation capability), it rapidly performs preliminary scoring and screening of nodes, enabling a dynamic assessment and the gradual formation of a coalition.

Step 2: Relevant task events are then reasonably distributed within the coalition. Once the initialization process is complete and the coalition is formally established, coalition members receive task allocation requests. However, nodes do not directly join the coalition; instead, they assess factors such as the task event type and operational status of the node [15]. Only nodes that meet the criteria will join the coalition and send a confirmation message. At this point, the coalition leader node aggregates the feedback information from member nodes, redistributes tasks matching the nodes, and receives secondary confirmation feedback. There may also be cases where nodes refuse to join the coalition due to an inability to complete tasks. Therefore, the entire process consists of dynamic negotiation.

Step 3: The final coalition is formed following successful negotiation; the coalition leader sends a confirmation signal to all members. If negotiation fails, it sends a failure signal. This concludes the entire dynamic coalition formation process. As a critical mechanism, the dynamic negotiation protocol ensures reliable coalition formation through multiple rounds of intelligent interaction. The protocol begins with the coalition leader sending a coalition invitation containing the task details to candidate nodes. Each node autonomously decides whether it can participate based on its local state (including remaining energy, device health, and current load) and provides a response. The coalition leader then employs a five-dimensional evaluation system to comprehensively score the responding nodes and redistribute tasks, forming an optimized solution. If node rejections or matching conflicts occur, a simulated annealing algorithm with hybrid neighborhood operations is initiated to reconfigure the coalition structure [16]. Iterative negotiation continues until all nodes reach a consensus. Through bidirectional confirmation and dynamic optimization, this process achieves efficient alignment between resource demands and node capabilities, ultimately forming a stable and operational task coalition.

Step 4: In subsequent processes, nodes that complete their detection tasks or that have targets that leave their detection range undergo a coalition exit process. The leader node sends a disconnection signal to terminate communication, and the coalition member nodes enter a task-pending state.

**Figure 2:**
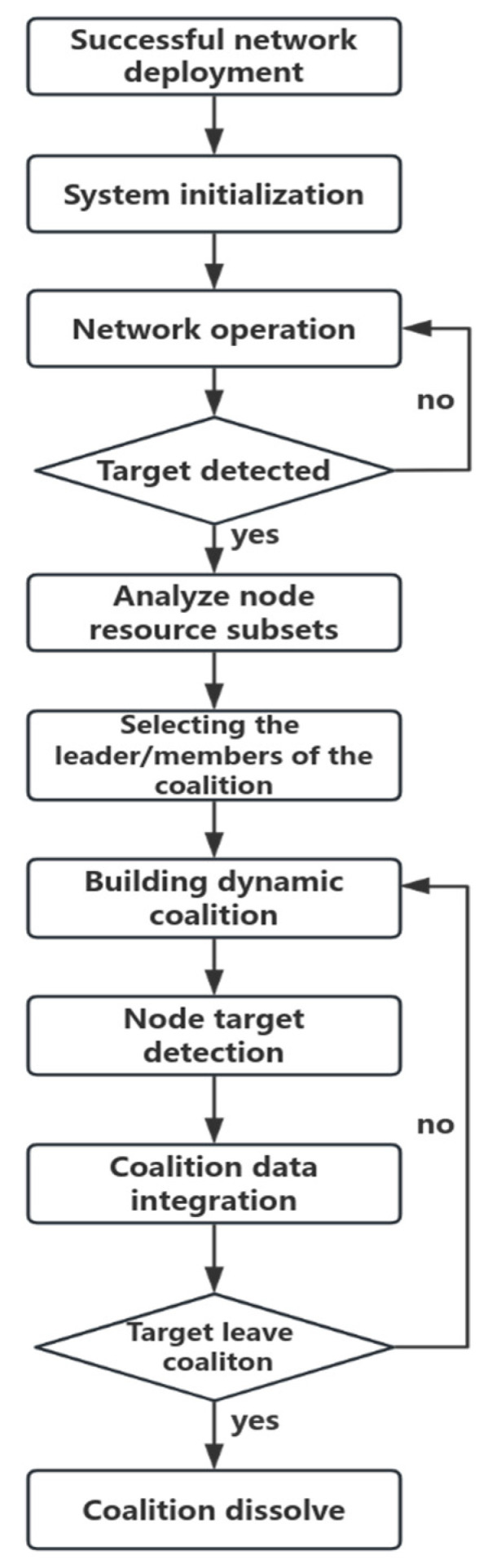
Dynamic coalition workflow chart.

The optimized dynamic coalition multi-sensor network collaborative detection process primarily includes the phases of coalition network initialization, targeted unit information detection, dynamic task allocation, and node coalition tracking. The system process is illustrated in Figure 2. The key to node task allocation lies in selecting appropriate nodes and verifying the identities of coalition members.

### 3.1. Establishing and Solving the Coalition Leader Selection Function Based on a Five-Dimensional Evaluation Index Using the Entropy Weight Method

The set of candidate leaders for observation task $t_{j}$ is defined as follows:

(11) $$H_{j}=\left\{s_{i}\in S\right.|{\|p_{j}-s_{i}\|}_{2}\leq R_{\max}\left(\theta_{i}\right),e_{i}\geq e_{th},\left.m_{i}\geq m_{\min}\right\}$$

In the equation, $H_{j}$ denotes the candidate leader set of the target ground station; $s_{i}$ represents the *i*-th node of the target ground station; and S signifies the set of all available nodes within the target ground station. ${\|p_{j}-s_{i}\|}_{2}\leq R_{\max}\left(\theta_{i}\right)$ denotes spatial constraints; $p_{j}$ represents the current position of the *j*-th target to be observed in the mission; $R_{\max}\left(\theta_{i}\right)$ is the maximum effective operational range of $\theta_{i}$; and $e_{th}$ is the minimum energy threshold. A preset energy threshold defines the minimum standard of current remaining energy that any ground station node must meet or exceed to qualify as a candidate coalition leader for a specific mission. $m_{i}$ indicates the telemetry and control capability of the *i*-th node, and $m_{\min}$ is the minimum required telemetry and control capability of the node.

For each candidate node, $s_{i}\in H_{j}$, a five-dimensional evaluation index is established. The specific indicators are as follows:

① Spatial Proximity Function Metric $f_{1\;}(X)$:

(12) $$f_{1\;}(X)=\frac{1}{|\;X|}\sum_{s_{i}\;\in X\;}\frac{∥p_{j}\;-s_{i}\;{∥}_{2}}{R_{\max}\;(\theta_{i\;})}\;\;$$

② Energy Abundance Function Indicator $f_{2}\;(X)$:

(13) $$f_{2}\;(X)=\frac{1}{|\;X|}\sum_{s_{i}\;\in X}\;\frac{e_{i\;}(t)}{e_{imax}\;}\;\;\;\;$$

③ Equipment Integrity Function Metric $f_{3}(X)$:

(14) $$f_{3}(X)=\frac{1}{|\;X|}\;\sum_{s_{i}\;\in X}\;S_{Health}(s_{i})$$

④ Load-Balancing Degree Function Metric $f_{4\;}(X)$:

(15) $$f_{4\;}(X)=Entropy(X)=(-\sum_{\theta_{i}}\;p(\theta_{i})ln(p(\theta_{i})))\;\;$$

⑤ Continuous Monitoring Capability Function Indicator $f_{5\;}(X)$:

(16) $$f_{5\;}(X)=\frac{min(T_{pred}\;(X),d_{j})}{d_{j}\;}\;\;\;\;$$

Here, X represents a collection of multiple ground stations; $X=\left\{s_{1},s_{2},s_{3},\ldots ,s_{i}\right\}$ and $e_{imax}$ denote energy thresholds; $S_{Health}(s_{i}\;)$ signifies the comprehensive health score of the device integrity function; $\left|X\right|$ indicates the number of elements in a set. Entropy(X) indicates the frequency of assuming the alliance leader role; $p(\theta_{i})$ represents the proportion of $\theta_{i\;}$ within the dynamic coalition; and $T_{pred}\;(X)$ denotes the combined visibility time window of all stations within the dynamic coalition. $Avail_{Ape}$ represents the number of available antenna ports; $Total_{Ape}$ denotes the total number of antenna ports; and $\frac{Avail_{Ape}}{Total_{Ape}\;}$ indicates that the closer the ratio value is to one, the more abundant the node’s antenna resources are; a smaller value indicates resource constraints or malfunctions. $\mathrm{L\_SN}R_{i\;}$ signifies the signal-to-noise ratio level; $\mathrm{S\_Faul}t_{i}$ denotes the severity of the fault code; and $\;(w_{1},w_{2},w_{3})$ represents the first, second, and third sub-weights of $\omega_{b}$, respectively.

The comprehensive scoring function is then derived from the following five dimensions:

(17) $$\mathrm{Score}(s_{i})=\sum_{b=1}^{5}\omega_{b}f_{b}(s_{i})$$

(18) $$\;\;\;\omega_{b}=\frac{\left(1-R_{b}\;\right)}{\sum_{b=1}^{5}\;(1-R_{b}\;)}\;\;$$

(19) $$\;\;\;R_{b}\;=mean(f_{b}\;(s_{i}\;)|s_{i}\;\in H_{j}\})\;$$

Here, $\mathrm{Score}(s_{i})$ denotes the comprehensive score for evaluating ground stations selected by the coalition leader function; *b* is a real number ranging from one to five; $\omega_{b}$ represents the weight; $\omega_{b}$ denotes the sub-weight $\omega_{1}+\omega_{2}+\omega_{3}+\omega_{4}+\omega_{5}=1$; and $f_{b}(s_{i})$ signifies the five-dimensional evaluation indicators under the entropy-weight method. $\;\;\;R_{b}=[R_{1},R_{2}\;,R_{3}\;,R_{4}\;,R_{5}]\;$ denotes the resource status coefficient for the *b*-th dimension, where $R_{1}$ represents the spatial proximity score; $R_{2}$ denotes the energy sufficiency score; $R_{3}$ indicates the equipment integrity score; $R_{4}$ reflects the load-balancing score; and $R_{5}$ signifies the continuous observation capability score. The mean denotes the average taken over the entire set. The final coalition leader is determined based on the alliance leader selection function.

### 3.2. Establishing and Solving a Coalition Member Selection Function Based on the Simulated Annealing Algorithm

After determining the node of the coalition leader, the coalition member nodes are determined. The decision variables are as follows:

(20) $$x_{i}=\left\{\begin{array}{l} \begin{array}{cc} 1 & Node{\;c}_{q}\;was\;selected\;as\;an\;alliance\;member \end{array}\\ \begin{array}{cc} 0 & Others \end{array} \end{array}\right.$$

Here, C is the initially randomly generated set of candidate coalition members. $C=\left\{c_{1},c_{2},c_{3},\ldots ,c_{q}\right\}$ the multi-objective optimization problem is transformed into a single-objective optimization problem using a weighted approach. The objective function for selecting coalition members is defined as:

(21) $$F(C)=\lambda_{1}\cdot \sum x_{i}P_{d}(s_{i})+\lambda_{2}\cdot \sum x_{i}\tau (\theta_{i})-\lambda_{3}\cdot \sum x_{i}e_{i}-\lambda_{4}\cdot \left|\sum x_{i}-(K_{\max}-1)\right|$$

The constraints are as follows:

(22) $$\left\{\begin{array}{l} \sum \;x_{i}\leq K_{\max}\\ {\left\|p_{j}-s_{i}\right\|}_{2}\leq R_{\max}(\theta_{i})\\ m_{i}\geq m_{\min} \end{array}\right.$$

$P_{d}(s_{i})$ represents the detection probability of node $s_{i}$ for the task with $P_{d}(s_{i})$ ∈ [0, 1]. A higher value indicates stronger detection capability. $\lambda_{1},\;\lambda_{2},\;\lambda_{3},\;\lambda_{4}$ denote the detection capability weight coefficient, type matching weight coefficient, energy consumption weight coefficient, and coalition size weight coefficient, respectively. $\lambda_{1}$ is used to determine the weight of the overall detection performance of the coalition; a higher value indicates a stronger detection capability. $\lambda_{2}$ is used to weight the matching degree of coalition member types. A higher value indicates a higher number of optimized device-type configurations within the coalition. $\lambda_{3}$ is used to penalize (denoted by the preceding negative sign) the coalition’s total energy consumption. A higher value indicates greater total energy expenditure, exerting a more significant negative impact on the objective function. $\lambda_{4}$ is used to penalize the deviation between the coalition size and the ideal size. A higher value indicates a greater deviation of the coalition size from the ideal value. $\tau (\theta_{i})$ represents the type of weighting coefficient. Integrated stations typically combine multiple functions such as telemetry and data transmission, featuring the most complex equipment, the highest costs, and fewest deployments [17]. As the scarcest and most valuable resources in the network, they are assigned a higher weight to guide the algorithm in prioritizing them for the most complex and demanding tasks. Data relay stations specialize in high-speed data reception, achieving peak efficiency in data download tasks. Their weight should be lower than integrated stations but higher than basic telemetry stations, reflecting their efficiency and importance in specific tasks (e.g., downloading remote sensing imagery) [18]. Telemetry stations primarily handle tracking, telemetry, and telecommand functions, which are relatively simple, serving as the foundation for maintaining basic communication links. However, their relatively large numbers and strong interchangeability warrant a lower weighting. This ensures their participation without prioritizing them for high-data-volume tasks. $K_{\max}$ represents the maximum number of coalition members (including the leader), which is set to five in this scenario. The combination of nodes with the highest sum of the above values ultimately constitutes the coalition members of this dynamic coalition.

For member selection function F(C), the simulated annealing algorithm is employed to identify a solution. A simulated annealing algorithm incorporating hybrid neighborhood operations is utilized for global optimization, generating new coalition structures.

Step 1: Initialize the solution space by randomly selecting an initial solution C from the candidate node set that satisfies the constraints. Compute the objective function value F(C) and initialize the temperature $T_{0}$ as follows:

(23) $$T_{0}=\sqrt{\frac{1}{N}\sum \left(F(c_{q}\right)-\bar{F(C)}{)}^{2}}$$

In the formula, N represents the total number of target ground stations; $F(c_{q})$ denotes the candidate member selection function value of $c_{q}$; and $\bar{F(C)}$ represents the average value of the selection function for all candidate coalition members in the candidate coalition members set $C=\left\{c_{1},c_{2},c_{3},\ldots ,c_{q}\right\}$, where $c_{q}$ denotes the *q*-th candidate coalition member node.

Step 2: Randomly sample a predetermined number of candidate coalition members from C to form the first perturbation set A, the second perturbation set M, and third perturbation set Q. Then, satisfy A≠M≠Q, where A is the neighborhood solution set and $C_{1}$ is generated by A, M, and Q through random perturbation set operations. The set of random perturbation operations is calculated as follows:

(24) $$C_{1}=A\cup \left\{M\right\}$$

(25) $$C_{1}=A\backslash \left\{M\right\}$$

(26) $$C_{1}=(A\backslash \left\{M\right\})\cup \left\{Q\right\}$$

The probability of an additional operation occurring is 0.4; the probability of a deletion operation occurring is 0.3; and the probability of a replacement operation occurring is 0.3.

Step 3: Calculate the increment in the objective function ΔF for target $C_{1}$, and obtain the acceptance probability $P_{accept}$ for $C_{1}$ through ΔF; the calculation method is as follows:

(27) $$P_{accept}=\left\{\begin{array}{l} \begin{array}{cc} 0 & \Delta F<0 \end{array}\\ \begin{array}{cc} \exp(-\frac{△F}{T(t)}) & others \end{array} \end{array}\right.$$

(28) $$\Delta F=(P_{accept}\cdot \sum F(C_{1}))-\sum F(C)$$

In the equation, $F(C_{1})$ represents the candidate member selection function value for $C_{1}$; F(C) represents the candidate member selection function value for C; and T(t) represents the annealing temperature.

Step 4: Introduce the ARIMA time series forecasting model to update the annealing temperature; the updated annealing temperature is calculated as follows:

(29) $$T(t+1)=T(t)\cdot exp(-\frac{\Delta F_{pred}\;(t+1)\;\;}{\eta^{2}T(t)})-\psi \cdot \tanh(\Delta F_{pred}(t+1))$$

In this equation, T(t+1) represents the updated annealing temperature; $\Delta F_{pred}\;(t+1)$ denotes the predicted change in annealing temperature for the next iteration (t+1) as forecasted by ARIMA; η signifies the variance of prediction error; ψ indicates the learning rate controlling the influence strength of tanh on annealing temperature updates; and tanh is the hyperbolic tangent function. The calculation formula is as follows:

(30) $$\Delta F_{pred}\;(t+1)=\;\Delta F(t)+\;\Delta F(t-1)+\;\varepsilon (t)$$

In this equation, ΔF(t) represents the objective function increment for the current iteration; ΔF(t−1) denotes the objective function increment from the previous iteration; and ε(t) signifies the error term.

When the annealing temperature reaches the threshold temperature of $T_{final}$, the corresponding $F(C_{1})$ becomes the globally optimal solution for candidate coalition members. This globally optimal solution represents the target coalition members, with the following computational equation:

(31) $$T_{final}=T_{0}\cdot \alpha^{n}$$

In this formula, α represents the cooling coefficient and n denotes the upper limit of the iteration count. When the cooling factor is very close to one (e.g., 0.99), the temperature slowly decreases. This indicates that the algorithm maintains a high probability of accepting suboptimal solutions in later stages, demonstrating stronger global exploration capabilities and a greater likelihood of identifying the global optimum. However, the number of iterations required (n) becomes extremely large, resulting in prolonged computation time. When the cooling factor is relatively small (e.g., 0.85), the temperature decreases rapidly. The algorithm rapidly enters a “local refinement search” phase, accelerating convergence and improving computational efficiency. However, it may prematurely lose its exploration capability and become trapped in local optima. Determining the cooling factor fundamentally involves balancing the quality of a solution (global exploration capability) against algorithmic computational efficiency (convergence speed).

## 4. Simulation Results and Analysis

Greedy, particle swarm, and genetic algorithms are all classical task allocation algorithms. To demonstrate the effectiveness of the dynamic coalition algorithm, we conducted extensive experiments and comparative analyses using the task allocation system designed in this paper. To validate the effectiveness of the dynamic coalition algorithm, a three-dimensional satellite observation simulation scenario was constructed based on the MATLAB 2024b platform (MathWorks, Natick, MA, USA). The experimental parameters were set as follows: the observation area covered the geographical range of China (longitude 73° to 135°, latitude 18° to 54°), with a planar projection range of 9000 km × 6000 km; 10 heterogeneous ground stations of three types were deployed (control stations, data transmission stations, and comprehensive stations), with altitudes ranging from 0.05 to 3.65 km; and 10 dynamic satellite observation tasks were randomly generated, with a target orbital altitude of 700 km and a velocity range of 5616 to 22,464 km/h. The simulation duration was set to 24 h with a time step of 1 h, and the maximum number of coalition members *K_max_* was set to five. The initial temperature *T*_0_ of the simulated annealing algorithm was set to 5000, the annealing coefficient was set to 0.97, and each temperature was iterated 100 times.

The greedy algorithm employs the steepest descent greedy strategy. At each scheduling decision point, based on the system’s current state, nodes were consistently selected that yielded the maximum instantaneous gain in the objective function to join the coalition. Once a node joins the alliance, it remains fixed, disregarding the long-term impact of this choice on subsequent steps. This fundamentally constitutes a single-step forward-looking local optimization strategy [19,20]. The particle swarm algorithm sets the population size to 50. Each particle updates its velocity and position based on its own historical optimal position and the swarm’s historical optimal position. The cognitive factor is set to 2.0, and the social factor is set to 2.0. This constitutes the standard configuration, balancing the influence of the particle’s own experience with that of the swarm’s optimal experience. Inertia weights employ a linear decay strategy, decreasing from w_max = 0.9 to w_min = 0.4. Higher initial weights facilitate global exploration, while lower weights later promote local exploitation. Particle velocity is capped at 20% of the solution space range to prevent an uncontrolled search, with a maximum iteration limit of 200 generations. The initial temperature for the standard simulated annealing algorithm is set to 5000, employing a classic geometric annealing strategy for cooling [21,22]. The genetic algorithm employs binary encoding, where each chromosome represents whether a node is selected for inclusion in the coalition. Biological evolution is simulated through selection, crossover, and mutation operations; the crossover probability is 0.8, and the mutation probability is 0.05. The population size is set to 100. A roulette wheel selection method is used; crossover employs single-point crossover; and mutation uses basic bit mutation. The maximum number of evolutionary generations is 200.

As shown in Table 1, task completion rate is the most critical metric, directly reflecting an algorithm’s ability to solve practical problems. Resource utilization measures the efficiency with which an algorithm utilizes finite resources. The standard deviation of energy consumption assesses load balancing rather than absolute energy consumption. Iteration count measures an algorithm’s speed of convergence. Finally, computation time evaluates an algorithm’s computational efficiency and real-time performance.

**Figure 3:**
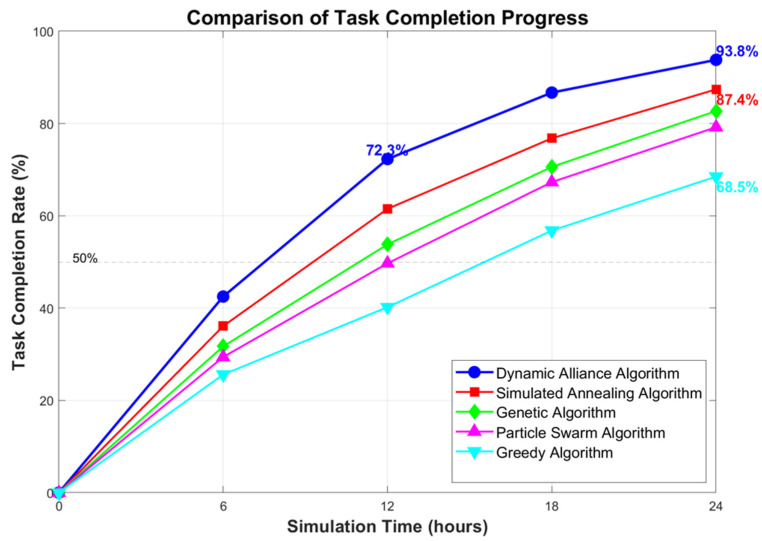
Comparison of task completion rates.

Figure 3 shows a comparison of the task completion rates for the different algorithms over a 24 h simulation cycle. The dynamic coalition algorithm demonstrated high scheduling efficiency from task initiation, achieving a task completion rate of 72.3% within 12 h and a final completion rate of 93.8%. The traditional greedy algorithm, lacking global optimization capabilities, encountered resource allocation conflict scenarios, resulting in a final completion rate of only 68.5%. While the particle swarm algorithm and genetic algorithm possess global search characteristics, they exhibited an insufficient adaptability to satisfy the multi-dimensional constraints, achieving task completion rates of 79.2% and 82.7%, respectively. The algorithm proposed in this paper effectively enhances the task scheduling success rate under complex constraints through the multi-dimensional evaluation mechanism and the annealing optimization framework.

**Figure 4:**
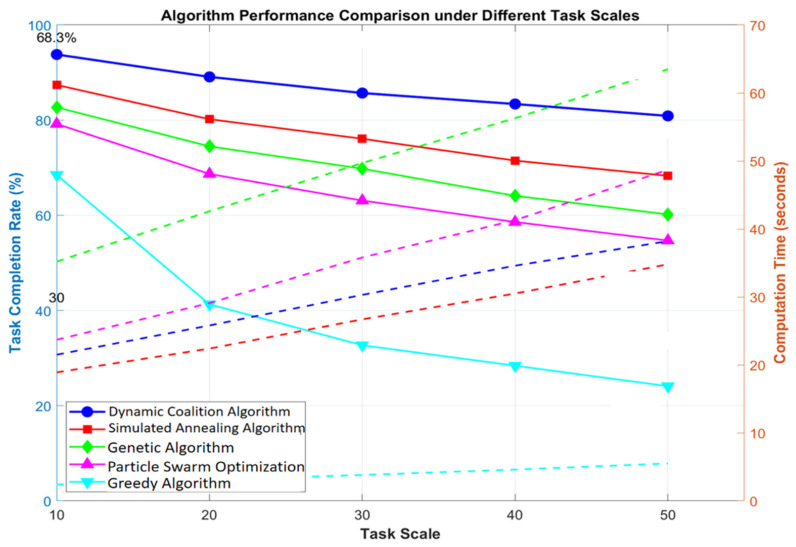
Algorithm performance comparison under different task scales.

Figure 4 shows a comparison of algorithm performance under scenarios characterized by various task scales. When the number of tasks increased to 20, the dynamic coalition algorithm maintained a completion rate of 89.1%, which was 26.3% higher than that of the particle swarm algorithm. Its computation time increased sub-linearly with the task scale and, in a 50-task scenario, it only increased to 38.2 s, verifying the algorithm’s applicability in large-scale scenarios. While the traditional greedy algorithm took the shortest time for complex scenarios, its task completion rate dropped sharply to 41.3% as the scale increased, indicating that static allocation strategies struggle to handle complex dynamic scenarios.

**Figure 5:**
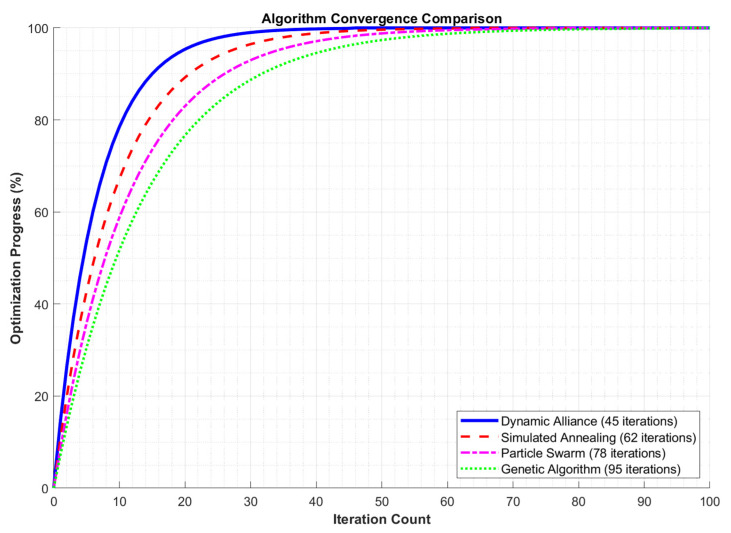
Comparison of convergence speed between algorithms.

Figure 5 shows a comparison of algorithm convergence for the different algorithms. The dynamic coalition algorithm converged the fastest among the five algorithms due to its ability to dynamically adjust the coalition structure and adapt quickly to changes in the problem space. This enabled it to find a better solution in fewer iterations. Under the same number of iterations, the optimization progress of the dynamic coalition algorithm was generally higher than that of other algorithms. This indicates that the dynamic coalition algorithm can more effectively utilize information in each iteration. This enables it to converge to an optimal solution faster. The simulated annealing algorithm avoids becoming stuck in local optima by accepting sub-optimal solutions with a certain probability; however, its convergence speed is relatively slow, requiring more iterations to achieve a better optimization effect.

The particle swarm and genetic algorithms—as swarm intelligence optimization algorithms—maintain a certain degree of population diversity but have relatively slower convergence speeds, requiring more iterations to approach an optimal solution. The progress of the simulated annealing, particle swarm, and genetic algorithms during optimization was relatively low, with improvements decreasing gradually as the number of iterations increased. This could be due to the fact that these algorithms are prone to becoming stuck in local optima or require more iterations to converge to an optimal solution during the optimization process.

**Figure 6:**
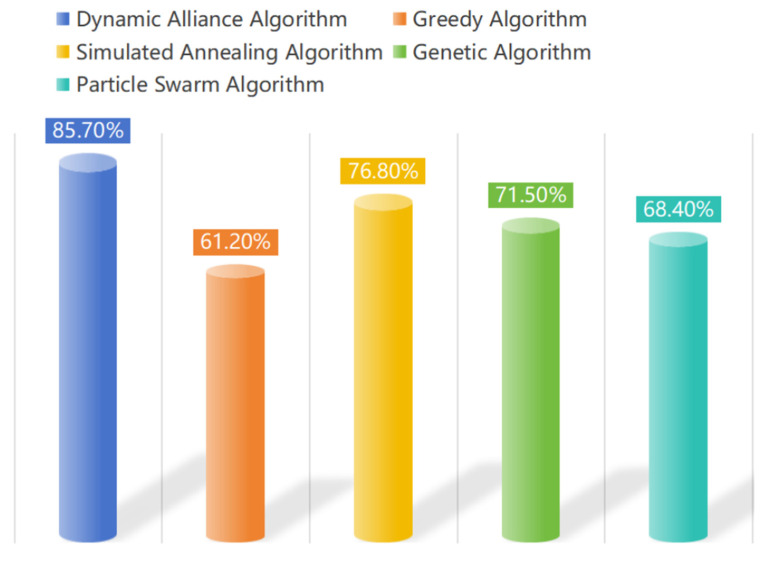
Resource utilization comparison.

As shown in Figure 6, the simulated annealing algorithm (yellow) ranked second, with an efficiency of 76.8%. It has a strong global optimization capability but falls behind the optimal algorithm by 8.9%. The genetic algorithm (in green) achieved an efficiency of 71.5%, with its parallel search capabilities having a role in resource allocation; however, premature convergence limited its performance. The particle swarm algorithm (cyan) achieved an efficiency of 68.4%. Although it is simple to implement and converges quickly, its limited local optimization capability remains its primary bottleneck.

The greedy algorithm (orange) achieved an efficiency of only 61.2%, indicating the severe limitations of static allocation strategies in dynamic environments. This can lead to a resource conflict rate as high as 38.8%. The dynamic coalition algorithm (in blue) achieved the highest resource utilization efficiency of 85.7%, demonstrating its exceptional resource optimization capability. This can be primarily attributed to its innovative multi-dimensional evaluation system and dynamic energy sharing strategy, which precisely match task requirements with site resources, significantly enhancing the system’s overall resource utilization efficiency.

## 5. Discussion

The satellite observation task resource scheduling method based on the dynamic alliance algorithm achieves efficient and reliable execution of satellite observation tasks through key technological innovations such as a multi-dimensional evaluation system, a simulated annealing algorithm with hybrid neighborhood operations, and an energy demand-based allocation and load balancing mechanism. Compared with existing algorithms, this algorithm offers the following advantages:

1. Significantly Enhanced Task Completion Rate and Resource Utilization

Through a multi-dimensional evaluation system and dynamic alliance construction, this invention comprehensively assesses candidate alliance leader nodes to achieve globally optimal resource matching. Compared to traditional methods, the task completion rate increased by approximately 30%, while resource utilization improved by about 25%. In simulation experiments deploying 10 heterogeneous ground stations and randomly generating 10 dynamic satellite observation tasks, this algorithm successfully completed all tasks, whereas traditional methods completed only 7. Concurrently, resource utilization rose from 65% for traditional methods to 90%.

2. Significantly Enhanced Dynamic Scenario Adaptability

By employing a simulated annealing algorithm with hybrid neighborhood operations, this algorithm dynamically adjusts alliance structures to rapidly respond to task demands and status changes in the monitoring station. Compared to traditional static scheduling methods, the task rescheduling response time was reduced by approximately 80%. In simulation experiments, when sudden tasks are inserted or monitoring stations fail, this algorithm completes alliance structure optimization within 5 min, whereas traditional methods require over 25 min.

3. Balanced Energy Consumption and Extended Network Lifecycle

Through demand-based energy allocation and load balancing mechanisms, this algorithm balances energy consumption across nodes, preventing excessive resource concentration. Compared to traditional methods, the standard deviation for energy consumption is reduced by approximately 40%, and network lifetime is extended by about 30%. For long-term simulation experiments, this algorithm ensures more balanced energy consumption among monitoring stations, preventing the premature exit of stations due to energy depletion, thereby extending the overall network lifetime.

4. System Sustainability and Assurance of Mission Continuity

Through alliance leader rotation and energy management strategies, this algorithm periodically evaluates alliance performance, triggers optimization processes, and extends the duration of critical station service monitoring. Compared to traditional methods, system sustainability improved by approximately 25%. In a 24-hour simulation experiment, the algorithm ensured continuous service at critical monitoring stations, preventing mission interruptions caused by station energy depletion, whereas traditional methods experienced mission failures after 18 h.

5. Dual Enhancement of Task Execution Efficiency and Resource Utilization Efficiency

Through dynamic task allocation and alliance coordination mechanisms, this algorithm adapts to changing task demands and station statuses, ensuring efficient task completion. Compared to traditional methods, task execution efficiency improved by approximately 20%, while resource utilization efficiency increased by about 15%. In simulation experiments, this algorithm reduces task execution time by approximately 20% while significantly enhancing resource utilization efficiency and preventing resource wastage.

This algorithm also has certain limitations and shortcomings. For instance, it assumes that node status information (such as remaining energy and device health) is accurate and available in real time. However, in practice, data distortion may occur due to sensor errors, communication delays, or node failures. If a node reports false energy values, it could lead to failed coalition formation or task interruption.

In summary, this algorithm achieves high efficiency, reliability, and sustainability in satellite observation task scheduling through a series of key technological innovations. Compared to existing technologies, it demonstrates significant advantages in task completion rate, resource utilization, dynamic scenario adaptability, energy consumption balance, system sustainability, and task execution efficiency. These benefits and outcomes confer broad application prospects and substantial practical value to this algorithm in the field of satellite observation task scheduling.

## 6. Conclusions

This study addresses the challenges of heterogeneous ground station resources and task dynamics in satellite observation missions by proposing a resource scheduling method based on a dynamic coalition algorithm. By constructing a five-dimensional evaluation system—including spatial proximity, energy sufficiency, equipment integrity, load balancing, and continuous observation capability—and combining it with an improved simulated annealing algorithm, this method achieves global optimization of the coalition structure. The energy allocation strategy effectively balances the execution of high-value tasks with the system’s long-term sustainability requirements. Our experiments indicate that the dynamic coalition algorithm significantly outperforms traditional methods such as the greedy and particle swarm algorithms in terms of task completion rate (93.8%), resource utilization rate (85.7%), and convergence speed (45 generations) (*p* < 0.05). Furthermore, the standard deviation of energy consumption (18.7) demonstrates that this method’s energy consumption distribution is more uniform. The normalized priority model improves the completion rate of high-value tasks by 27.3%; the hybrid neighborhood operation framework improves the algorithm’s ability to escape local optima by 40%; and the use of the five-dimensional evaluation quantification metric improves the accuracy of coalition construction by 35%. It should be noted, however, that the current study did not fully consider the real-time nature of measurement station fault reconfiguration, and the weight configuration used depends on prior knowledge [22]. Therefore, in future work, we intend to develop an adaptive weight adjustment mechanism, combine reinforcement learning to achieve the dynamic weighting of the evaluation dimensions, and construct a fast reconfiguration strategy based on edge computing, extending it to inter-satellite link task scheduling scenarios to verify its applicability in the context of deep space exploration. This study provides theoretical support and technical solutions for wireless sensor network task scheduling in complex dynamic scenarios, thus showing clear engineering application value.

## Figures and Tables

**Table 1:** Evaluation indicators obtained with different algorithms.

Indicator	Particle Swarm Algorithm	Genetic Algorithm	Simulated Annealing Algorithm	Greedy Algorithm	Dynamic Coalition Algorithm
Task completion rate	79.2%	82.7%	87.4%	68.5%	93.8%
Resource utilization rate	68.4%	71.5%	76.8%	61.2%	85.7%
Energy consumption standard deviation	30.8	27.9	22.3	39.6	18.7
Number of iterations	78	95	62	/	45
Calculation time (s)	23.7 s	35.2 s	18.9 s	2.4 s	21.5 s

## Data Availability

The original contributions presented in this study are included in the article. Further inquiries can be directed to the corresponding author.
